# Genetic Variants Shaping Inter-individual Differences in Response to Dietary Intakes—A Narrative Review of the Case of Vitamins

**DOI:** 10.3389/fnut.2020.558598

**Published:** 2020-12-01

**Authors:** Aikaterini Niforou, Valentini Konstantinidou, Androniki Naska

**Affiliations:** ^1^Department of Hygiene, Epidemiology and Medical Statistics, School of Medicine, National and Kapodistrian University of Athens, Athens, Greece; ^2^DNANUTRICOACH®, MEDOLIALI SL, Barcelona, Spain

**Keywords:** genes, diet, nutrigenetics, nutritional status, SNPs, genetic variants, vitamins

## Abstract

Recent advances in the field of nutrigenetics have provided evidence on how genetic variations can impact the individuals' response to dietary intakes. An objective and reliable assessment of dietary exposures should rely on combinations of methodologies including frequency questionnaires, short-term recalls or records, together with biological samples to evaluate markers of intake or status and to identify genetic susceptibilities. In an attempt to present current knowledge on how genetic fingerprints contribute to an individual's nutritional status, we present a review of current literature describing associations between genetic variants and levels of well-established biomarkers of vitamin status in free-living and generally healthy individuals. Based on the outcomes of candidate gene, genome-wide-association studies and meta-analyses thereof, we have identified several single nucleotide polymorphisms (SNPs) involved in the vitamins' metabolic pathways. Polymorphisms in genes encoding proteins involved in vitamin metabolism and transport are reported to have an impact on vitamin D status; while genetic variants of vitamin D receptor were most frequently associated with health outcomes. Genetic variations that can influence vitamin E status include SNPs involved in its uptake and transport, such as in SCAR-B1 gene, and in lipoprotein metabolism. Variants of the genes encoding the sodium-dependent vitamin C transport proteins are greatly associated with the body's status on vitamin C. Regarding the vitamins of the B-complex, special reference is made to the widely studied variant in the MTHFR gene. Methodological attributes of genetic studies that may limit the comparability and interpretability of the findings are also discussed. Our understanding of how genes affect our responses to nutritional triggers will enhance our capacity to evaluate dietary exposure and design personalized nutrition programs to sustain health and prevent disease.

## Introduction

The assessment of the population's nutritional status lies in the heart of health monitoring surveys and epidemiological studies aiming to elucidate diet-disease associations. Methods employed have traditionally relied on questionnaires, food diaries as well as markers assessed in biological samples (1). The thus accumulated evidence has enriched our understanding of the complex interplay among nutrients and other food substances; patterns of dietary intake; and, lifestyle and environmental exposures that shape an individual's nutritional status. Furthermore, advances in the field of nutrigenetics illustrate how genetic variations can impact on an individual's response to a nutritional trigger (i.e., a dietary intake) and, possibly, on the risk of nutrition-related diseases (2). It has now been generally recognized that objective dietary assessment should not solely rely on one method, but should employ combinations of methodologies including participants' self-reporting, as well as biological samples to assess established biomarkers and to identify routes of genetic susceptibilities.

In an attempt to present current knowledge on how genetic fingerprints participate in shaping the body's vitamin status, we have conducted a narrative review of studies evaluating associations between genetic variants and levels of well-established biomarkers of status of vitamin D, tocopherols and tocotrienols (vitamin E), vitamin C and vitamins of the B-complex, namely folic acid (B9) and cobalamin (B12), for which there is adequate evidence relating genetic variants and body status in free-living and generally healthy individuals.

## Methods

### Studies Identification and Selection

We performed an online literature search in PubMed until March 2020. The search terms used included one term related to the vitamins under study (i.e., vitamin D; tocopherol, vitamin E; ascorbic acid, vitamin C; folic acid, folate, vitamin B9; and cobalamin, vitamin B12) followed by the term “status” and combined with either “nutrigenetics” or “polymorphisms.” A study was considered if: (a) it was conducted among free-living healthy individuals and (b) was available in English. Original research papers, systematic reviews, and meta-analysis were all combined. In addition, reference lists of identified publications were further screened to identify additional literature, with the application of citation chasing techniques including reference list scanning of included studies and previous reviews, as well as backward and forward references of included studies. No limits on geographical location were applied.

Based on the search criteria and keywords used, 848 articles were identified. Among these, 817 articles were excluded after either title/abstract or full text screening since they did not address the aim of the present review, i.e., to collectively report on evidence regarding genetic variants that could impair the body's vitamin status. Finally, 31 articles were initially considered and through reference screening, we identified 30 additional publications. Overall, 60 articles have been considered in this review (Table 1).

**Table 1:** Characteristics of the publications included in the review (*n* = 60).

**References**	**Type of study**	**Reference to vitamin**	**Study participants (original research articles) or number of publications in meta-analyses**
Ahn et al. (3)	GWAS	Vitamin D	Five cohorts of 4,501 persons of European ancestry
Andrew et al. (4)	Cross-sectional (Twin Study)	Vitamin B12	*n* = 2,110 female twins of women of North European descent (Caucasian), 18–79 y
Bahrami et al. (5)	Review	Vitamin D	
Batai et al. (6)	Cross-sectional	Vitamin D	*n* = 1,057 (*n* = 652 African Americans, *n* = 405 European Americans), US
Bendik et al. (7)	Review	Vitamin D	
Block et al. (8)	Cross-sectional	Vitamin C	*n* = 383 non-smokers Caucasians 18–85 y (participants in previous RCT)
Bhan (9)	Review	Vitamin D	
Borel et al. (10)	Cross-sectional	Vitamin E	*n* = 128, Caucasians 18–70 y (participants enrolled in the Medi-RIVAGE study)
Borel Desmarchelier (11)	Review	Vitamin E	
Bu et al. (12)	Cross-sectional (CGAS)	Vitamin D	*n* = 496, non-Hispanics
Bueno et al. (13)	Cross-sectional	Folate	*n* = 726, Spanish adults 18–75 y
Cahill et al. (14)	Cross-sectional	Vitamin C	*n* = 905 non-smoking subjects, 20–29 y (participants in the Toronto Nutrigenomics and Health Study)
Cahill and El Solemy (15)	Cross-sectional	Vitamin C	*n* = 1.277 non-smoking subjects, 20–29 y (participants in the Toronto Nutrigenomics and Health Study)
Cahill and El Solemy (16)	Cross-sectional	Vitamin C	*n* = 1,046 non-smoking subjects, 20–29 y (participants in the Toronto Nutrigenomics and Health Study)
Castro et al. (17)	Cross-sectional	Vitamin B12	*n* = 122 Portuguese individuals (mean age 46 y)
de Batlle et al. (18)	Cross-sectional	Folate	*n* = 988 French women 40–65 y (participants in French E3N-EPIC cohort)
Duan et al. (19)	Systematic Review and meta-analysis	Vitamin D	Sixteen publications with a total of 52,417 participants of Asian and Caucasian origin
Duarte et al. (20)	Cross-sectional	Vitamin C	*n* = 80 Brazilian subjects
Duell et al. (21)	Case-control (nested within EPIC Cohort)	Vitamin C	*n* = 1,649 subjects, 57–70 y
Galmés et al. (22)	Review	Vitamin E	
Grarup et al. (23)	GWAS	Vitamin B12	*n* = 45,574 Icelandic and Danish individuals
Engelman et al. (24)	Cross-sectional	Vitamin D	*n* = 1,379 participants of 40 y mean age (from IRAS Family Study), Hispanics and African Americans
Ferrucci et al. (25)	GWAS	Vitamin E	*n* = 3,941 Caucasians (participants in InCHIANTI, WHAS, and ATBC studies)
Hansen et al. (26)	Cross-sectional	Vitamin D	*n* = 989, African Americans, 70–79 y
Hazra et al. (27)	Meta-analysis	Vitamin B12	*n* = 4,763, Caucasians (from three GWA scans)
Herrmann et al. (28)	Review	Vitamin D	
Hiraoka and Kagawa (29)	Review	Folate	
Horska et al. (30)	Cross-sectional	Vitamin C	*n* = 388 Slovakian factory workers
Hustad et al. (31)	Cross-sectional	Folate	*n* = 10,601 adults 50–64 y (from the Norwegian Colorectal Cancer Prevention cohort)
Jolliffe et al. (32)	Review	Vitamin D	
Kwak et al. (33)	Cross-sectional	Vitamin D	*n* = 2,264, Korean adults >20 y
Lauridsen et al. (34)	Cross-sectional	Vitamin D	*n* = 595 Danish Caucasian postmenopausal women (participants in DOPS Study)
LeCompte et al. (35)	Cross-sectional	Vitamin E	*n* = 1,614 Caucasian adults (participants in SU.VI.MAX and HELENA studies)
Major et al. (36)	GWAS	Vitamin E	*n* = 5,006 men of European descent (in two adult cohorts)
Major et al. (37)	GWAS	Vitamin E	*n* = 2,112, Finnish male smokers, 50–69 y (participants in the ATBC trial)
McGrath et al. (38)	Review	Vitamin D	
McNulty et al. (39)	Cross-sectional	Folate	*n* = 123, Irish individuals, 19–63 y
McNulty et al. (40)	RCT	Folate	*n* = 89 Irish individuals, 18–65 y
Michels et al. (41)	Review	Vitamin C	
Molloy (42)	Review	Folate	
Na et al. (43)	Cross-sectional	Vitamin C	*n* = 110 Chinese individuals, 18–50 y
Orton et al. (44)	Longitudinal population-based twin study	Vitamin D	*n* = 178 Canadian twins, 34–78 y
Robien et al. (45)	Cross-sectional	Vitamin D	*n* = 504, Singapore Chinese men and women, 45–74 y
Schwartz (46)	Review	Vitamin C, Folate	
Shane (47)	Review	Folate	
Shane et al. (48)	GWAS analysis	Folate	*n* = 2,232 Irish subjects, 18–28 y
Signorello et al. (49)	Cross-sectional	Vitamin D	*n* = 758 (*n* = 379 African Americans, *n* = 379 Caucasians), 40–79 y
Stanislawska-Sachadyn et al. (50)	Cross-sectional	Vitamin B12	*n* = 613 Northern Irish Men (Caucasians) 30–49 y
Surendran et al. (51)	Review	Vitamin B12	
Thuesen et al. (52)	Cross-sectional	Vitamin B12Folate	*n* = 6,784 Danish, 30–60 y
Timpson et al. (53)	Meta-analysis	Vitamin C	Five cohorts with a total of 15.087 Caucasians
Tsang et al. (54)	Systematic Review and meta-analysis	Folate	Forty publications with non-pregnant, non-lactating females 12–49 y across various population groups
Touvier et al. (55)	Cross-sectional	Vitamin D	*n* = 1,828, Caucasian adults, <65 y
Voipio et al. (56)	Cross-sectional	Vitamin D	*n* = 2,204, Finnish subjects, 30–45 y (participants in prospective Cardiovascular Risk in Young Finns Study)
von Castel-Dunwood et al. (57)	Cross-Sectional	Vitamin B12	*n* = 344 non-pregnant women, 20–30 y, non-Hispanic white (93%), non-Hispanic black (7%)
Wang et al. (58)	GWAS	Vitamin D	~30,000 (*n* = 33.869) Individuals of European descent from 15 cohorts.
Wright et al. (59)	Case-control (nested within the ATBC Trial)	Vitamin E	*n* = 1,833 (982 cases, 851 controls), Finnish male smokers, 50–69 y
Xu et al. (60)	Cross-sectional	Vitamin D	*n* = 1,873 (*n* = 945 Uygur/*n* = 928 Kazak ethnic)
Zanon-Moreno et al. (61)	Case-control study	Vitamin C	*n* = 300 (150 cases, 150 controls) Caucasian participants, 50–80 y
Zinck et al. (62)	Cross-sectional	Vitamin B12	*n* = 3,114 Canadians (85% Caucasians, 15% non-Caucasians) 20–79 y

## Vitamin D

Vitamin D is a fat-soluble vitamin that regulates bone growth and health through promotion of calcium absorption and maintenance of adequate calcium and phosphate concentrations. Vitamin D is further involved in the cell growth process and neuromuscular and immune activities (63, 64).

Vitamin D exists in two main forms: ergocalciferol (D2) and cholecalciferol (D3). Vitamin D3 is naturally present in animal foods (e.g., fatty fish, fish liver oils, dairy, egg yolk) and vitamin D2 is naturally present in some higher fungi. Vitamin D3 can also be synthesized endogenously in the skin from its provitamin 7-dehydrocholesterol, following exposure to UV-B radiation and subsequent thermal isomerization. The cutaneous synthesis in the human body is the main source of vitamin D3 and varies depending on the individual's exposure to sun, age, skin color, time spent outdoors, use of sunscreen, as well as the season of the year (64).

### Vitamin D Metabolism

In the body, dietary or endogenous vitamin D (D2 and D3) is either converted into its biologically active metabolite, following two hydroxylations, or transferred to the storage tissues. Activation of vitamin D involves two steps. The first occurs in the liver, where vitamin D is hydroxylated to 25-hydroxyvitamin D, 25(OH)D, also known as calcidiol. The 25(OH)D attaches to the vitamin D binding protein (DBP) and is transported to the kidneys where it is hydroxylated to form the 1,25-dihydroxyvitamin D, 1,25(OH)_2_D, also known as calcitriol. A mitochondrial enzyme (CYP27A1) and several microsomal enzymes (including CYP2R1) play an important role in the process of the 25-hydroxylation of vitamin D in the liver. Vitamin D, in the form of either 1,25(OH)_2_D or 25(OH)D, is transported in the blood primarily bound to the DBP. Upon its release, the biologically active form 1,25(OH)_2_D reaches the main target tissues including the intestine, the kidneys, and the bone, where it binds to the intracellular vitamin D receptor (VDR). After hydroxylation of vitamin D in the liver, serum 25(OH)D is also delivered to the adipose tissue, muscle, and liver for long-term storage (65). Metabolites of both 25(OH)D and 1,25(OH)_2_D are degraded in an oxidative pathway mediated by the CYP24A1 inactivation protein (24-hydroxylase) (64, 66).

### Biomarkers of Vitamin D Status

The intake through diet and the level of exposure to sun are the most important determinants of vitamin D status. Serum concentration of 25(OH)D indicates the overall vitamin D levels derived from both cutaneous synthesis and dietary sources. It is considered a reliable marker of vitamin D status with a half-life of ~13 to 15 days because of its strong affinity for DBP (63, 67). In populations with low exposure to UV-B radiation, serum 25(OH)D can also be used as a biomarker of intake (68). However, the individual variability observed in vitamin D status could be attributed to different analytical methods, the use of distinct reference values to assess the body status and to genetic factors (28). Thus, assessments of vitamin D status in population studies should be interpreted with caution (64).

### Vitamin D Deficiency

Taking into account risk for rickets or symptomatic osteomalacia, serum 25(OH)D levels below 25 nmol/L have been suggested as an indicator of vitamin D deficiency in Europe (64). The Institute of Medicine concluded that people with serum concentrations below 30 nmol/L and between 30 and 50 nmol/L are susceptible to vitamin D deficiency and inadequacy, respectively (63). The Endocrine Society Task Force suggests that 25(OH)D concentration below 50 nmol/L indicates vitamin D deficiency (69). In general, a threshold of less than 25–30 nmol/L characterizes vitamin D deficiency, but to date a standard definition regarding the “optimal” 25(OH)D levels is still lacking (70). Recently, a target population value of 50 nmol/L for serum 25(OH)D concentration is proposed by EFSA (64).

### Genetic Variations and Vitamin D Status

Genetic variants of proteins participating in the vitamin D metabolism, its binding to receptors and transport can have an impact on vitamin D availability and status (5, 7). Several single nucleotide polymorphisms (SNPs) related with serum 25(OH)D that have been detected in candidate gene studies and genome-wide association studies (GWAS) have enhanced our understanding of vitamin D balance; the detection of individuals more vulnerable to deficiency; as well as those who could benefit more than others from supplementation (5).

The Study of Underlying Genetic Determinants of Vitamin D and Highly Related Traits (the SUNLIGHT study) is the largest GWAS published to date that identified significant genetic determinants of 25(OH)D levels. The study involved individuals of European descent from 15 cohorts (*n* = 33,996) and reported variants at three loci reaching genome-wide significance in both discovery and replication cohorts for association with 25(OH)D concentrations. The genetic variants identified in this large study belong to the genes encoding DBP (GC gene), enzyme 7-dehydrocholesterol reductase (DHCR7 gene), vitamin D 25-hydroxylase (cytochrome P450, family2, subfamily R, member 1, CYP2R1 gene), and vitamin D 24-hydroxylase (cytochrome P450, family 24, subfamily A, member 1 CYP24A1 gene) (58) and confirm the results of previous smaller-scale studies (12). With respect to variants of the gene encoding the intracellular VDR, there are no consistent findings relating to vitamin D status. In a recent systematic review, Jolliffe et al. (32) reported that polymorphisms in DBP, CYP2R1, and DHCR7 are the ones most frequently associated with 25(OH)D blood concentrations, while variants in the VDR gene have mostly been reported as determinants of phenotypes. Genes implicated in vitamin D metabolism and signaling pathways have been schematically presented by Jolliffe et al. (32).

### Vitamin D Binding Protein Related Variants (DBP)

The DBP initially known as Gc-globulin (group-specific component of serum), is a circulating alpha globulin produced primarily by the liver at relatively stable levels throughout life, except from conditions of high estrogen levels (e.g., during pregnancy, when production is increased) The DBP is a multi-functional compound that is involved in the binding of the majority (>85%) of circulating 25(OH) D, and also of extracellular actin and in the transport of fatty acids (71). The DBP has the highest affinity for 25(OH)D-lactones, followed by 25(OH)D and 1,25(OH)_2_D (72) and binds more effectively to vitamin D3 and its metabolites than vitamin D2 and its metabolites (73). Variants of the DBP gene have been widely investigated as sources of variation in the circulating levels of vitamin D (5, 32).

The GC gene encodes the DBP and is located on chromosome 4q12-q13. The two most frequently investigated variants in the GC gene that modify the amino acid sequence of the protein are the SNPs found in exon 11. The rs4588 DBP SNP causes an amino acid change from lysine to threonine in codon 420 (substituting A for C, ACG → AAG, Thr → Lys) resulting in a Gc-2 protein. In addition, the rs7041 SNP leads to a change of aspartate to glutamate in codon 416 (substituting G for T, GAT → GAG, Asp → Glu) resulting in a Gc-1s protein (9). Although over 120 variants in the DBP gene have been recognized, three main phenotypic alleles-GC haplotypes (Gc1S, Gc1F, and Gc2) have been identified. Regarding electrophoretic migration, the slowest is Gc2, followed by Gc1S (slow) and Gc1F (fast) (74). The associated serum concentration of DBP and its affinity toward 25(OH)D differ among the phenotypic alleles (75, 76). The median concentration of 25(OH)D is the highest in Gc1s-1s (CC rs4588; GG rs7041), intermediate in Gc1-2 (Gc1F) (CC rs4588; TT rs7041), and lowest in Gc2-2 (AA rs4588; TT rs7041) (28, 34). These haplotypes further exhibit an important racial and geographic variation, since GC1F is more prevalent among dark-skinned individuals (particularly those of African descent), while Gc1S and Gc2 are more common in Caucasians (77, 78). The association between the rs4588/rs7041 SNPs with serum 25(OH)D levels has been confirmed in studies including different ethnic populations (32). Minor A allele of rs4588 is consistently associated with lower 25(OH)D levels, while minor G allele of rs7041 with higher 25(OH)D levels (32). In Caucasian adults, these associations were found to be significant after adjustment for vitamin D dietary intake, sun exposure, socio-demographic, anthropometric, and lifestyle factors (*P* < 0.0001) (55). Furthermore, in a recent study among African Americans the T allele of rs7041 was associated with lower serum 25(OH)D (b = −0.93, SE = 0.53, close to significance thresholds, *P* = 0.08) (26). Individuals with the Gc2-2 haplotype (AA rs4588 /TT rs7041) have significantly lower 25(OH)D concentrations compared to all other Gc haplotypes (*p*-trend < 0.001). In addition, individuals with Gc1s-1s haplotype (CC rs4588/GG rs7041) are characterized by higher 25(OH)D status (45). These findings confirm earlier observations between plasma 25(OH)D concentrations and Gc phenotypes reported by Lauridsen et al. (34).

Two GWAS meta-analyses identified another polymorphism in the GC gene (the rs2282679) which is located within intron 12 (A>C) and is in a high linkage disequilibrium with rs4588 (3, 58). In the Ahn et al. (3) meta-analysis of five cohorts of individuals with European ancestry, the (minor) risk C allele has been found to be inversely associated with circulating vitamin D levels (b = −0.36, SE: 0.05). Per copy of the minor allele of this variant, individuals have 49% higher risk of vitamin D insufficiency [<50 nmol/L OR = 1.49, 95% CI = 1.40–1.59, *P* = 7.5 × 10^−33^], after adjusting for age, sex, BMI, and season (58). A stronger association between the risk allele and vitamin D insufficiency has also been reported by Voipio et al. (56) in a study among Finish adults (OR = 2.08, 95% CI = 1.66–2.60). In a study among European Americans, the C allele of rs2282679 was associated with lower circulating 25(OH)D levels (b = −0.05, *p* = 0.001) (6). In studies among Koreans (33) and African-Americans (49) individuals carrying the G minor allele had lower 25(OH)D concentrations, as compared to those carrying the T allele.

### 7-Dehydrocholesterol Reductase Related Variants

Cholecalciferol and cholesterol are synthesized through the skin formation of 7-dehydrocholesterol (7-DHC). The DHCR7 gene on chromosome 11 encodes the enzyme 7-dehydrocholesterol reductase, which converts 7-DHC to cholesterol, reducing thus the availability of this precursor (7-DHC) for the synthesis of vitamin D (5). The UV-B radiation and the enzyme possibly antagonize in the conversion of 7-DHC and as a result, 25(OH)D has been reported to have a varying association with serum HDL or skin cholesterol levels shaped by the interrelation between the DHCR7 enzyme in serum and the amount of sun exposure (79).

Several studies have shown that the DHCR7 variant (rs12785878) is associated with 25(OH)D concentrations (overall *p* = 2.1 × 10^−27^, combined discovery and replication samples) (58) The rs12785878 variant is located in an intron of a different gene known as NADSYN1 and as a result this SNP is often indicated as the NADSYN1/DHCR7 locus. Homozygotes (GG) have lower mean 25(OH)D levels than heterozygotes (GT), who in turn on average have lower mean 25(OH)D levels than major homozygotes TT (58). A study among 2.204 Finnish individuals confirmed the association of the G minor allele of rs12785878 with lower 25(OH)D concentrations (b = −2.10, SE = 1.01) and the increased risk of vitamin D insufficiency (<50 nmol/L, OR = 1.31, 95% CI = 1.00–1.70) compared to the T allele and after adjusting for vitamin D intake, nutrient supplement use, body mass index, physical activity, and lifestyle factors (56). In a multi-center study including individuals of Kazak ethnicity, the DHCR7/NADSYN1(rs12785878) has also been significantly associated with vitamin D deficiency (25(OH)D levels <20 ng/mL) (OR = 2.44, 95% CI = 1.22–4.87), adjusted for sex, age, BMI, and study center (60). The negative association between G allele of rs12785878 (DHCR7) and 25(OH)D levels was also reported in a study of healthy Korean adults (33).

### CYP2R1 & CYP24A1 Related Variants

The human CYP2R1 gene (on chromosome 11p15.2) encodes a member of the cytochrome P450 superfamily of enzymes, the microsomal vitamin D 25-hydroxylase, involved in vitamin D activation through hydroxylation, to the vitamin D receptor ligand. The CYP24A1 (on chromosome 20q13.2) encodes the vitamin D 24-hydroxylase, participating in the inactivation process of vitamin D metabolites. Several GWAS have identified SNPs in the CYP2R1 and CYP24A1 genes associated with 25(OH)D concentrations (i.e., rs10741657, rs1993116, rs12794714, and rs10766197 for CYP2R1 and rs6013897 for CYP24A1) (32).

The rs10741657 (G>A) SNP is located in the non-coding region 5′-UTR, which may regulate gene expression and therefore modulate the expression and activity of 25-hydroxylase. In a large GWAS, Wang et al. (58) reported an association between the rs10741657 polymorphism and the 25(OH)D levels (overall *p* = 3.3 × 10^−20^, combined discovery and replication samples). In their recent meta-analysis, Duan et al. (19) confirmed this association and further reported that the GG genotype was associated with lower 25(OH)D levels when compared with the AA (reference) genotype [standardized mean difference SMD = −2.31, 95% CI = (−4.42, −0.20) overall and SMD = −3.46, 95% CI = (−6.60, −0.33) and SMD = −0.24, 95% CI = (−0.51, −0.03) for Caucasian and Asian groups, respectively]. Consequently, the risk G allele has been associated with an increased risk of vitamin D deficiency (<20 ng/mL or 50 nmol/L) compared to no-risk allele A in both Caucasian and Asian populations (OR = 1.09; 95% CI = 1.03–1.15). Under the dominant model (GG+AG vs. AA), Duan et al. (19) reported a 42% higher risk of vitamin D deficiency (95% CI = 11–83%); nevertheless, under the recessive model (GG vs. AG+AA), the positive association remained but lost significance (OR = 1.28; 95% CI = 0.89–1.84).

Among African-Americans, the strongest association with 25(OH)D levels has been observed for the CYP2R1 rs12794714 variant located in exon 1 (G>A). The minor (A) allele has been significantly associated with lower 25(OH)D levels (b = −0.4, *p* = 0.01) (6), a finding also reported by Wang et al. (58) (overall *p* = 2.7 x 10^−9^). Among European-Americans the most relevant determinant of vitamin status was the CYP2R1 rs1993116 SNP, located in intron 1 (C>T) with the minor A allele was found to be associated with higher serum 25(OH)D levels (b = 0.04, *p* = 0.0006) (6). This association has also been observed in two large GWAS by Wang et al. (58) (overall *p* = 6.3 x 10^−11^) and Ahn et al. (3) (risk A allele b = 0.25, *p* = 2.9 x 10^−17^). Associations of CYP2R1 rs12794714 and rs1993116 variants with vitamin D status have also been found significant in Chinese subjects (45). Furthermore, the rs10766197 variant, located in the promoter of the CYP2R1 gene has been associated with the 25(OH)D levels in the discovery cohort of healthy Caucasian subjects and remained significant after further replication and analysis of the pooled dataset and after adjusting for age, sex, BMI, habitual vitamin D supplementation and season. The minor A allele of rs10766197 has been inversely associated with serum 25(OH)D levels (b = −4.53, adjusted empirical *P*-value = 0.002, based on the pooled dataset analysis) (12).

A genetic risk score (GRS) was calculated by Wang et al. (58) combining three confirmed genetic variants related to circulating vitamin D levels (i.e., DHCR7 rs12785878; the CYP2R1 rs10741657; and, the GC rs2282679). Individuals with a “genotype score” in the top quartile had a 2-fold higher odds of vitamin D insufficiency (25(OH)D levels below 50 nmol/L) in comparison to the lowest quartile (OR = 1.92, 95% CI = 1.70–2.16, *P* ≤ 1 × 10^−26^)]. After adjusting for age, sex, body mass index, and season, the odds of vitamin D deficiency (i.e., 25(OH)D levels below 20 nmol/L) increased by 43% (adjusted OR = 1.43, 95% CI = 1.13–1.79) among individuals in the top quartile of this score in comparison to those in the lowest quartile. In the same study, Wang et al. (58) further reported that the rs6013897 SNP was also associated with serum 25(OH)D concentrations (overall *p* = 6 x 10^−10^ combined discovery and replication samples).

### The VDR Related Variants

Two VDR SNPs—the rs2228570 and rs10783219—have been associated with serum 25(OH)D concentrations. Both the rs2228570 and the rs10783219 SNPs have been associated with lower 25(OH)D levels in a longitudinal population-based twin study and in a sub-population of a cross sectional family study, respectively (24, 44). In general, the way in which the VDR gene variants could influence the 25(OH)D concentrations (38) has not yet been elucidated; in their review of genetic association studies, Jolliffe et al. (32) suggested that genetic variation in VDR is strongly related to the phenotype rather than circulating 25(OH)D concentrations.

## Vitamin E

Vitamin E is a fat-soluble vitamin, existing in biologically different forms, the tocopherols (α, β, γ, δ) and the tocotrienols (α, β, γ, δ), that possess different antioxidant activity. In humans, α-tocopherol is the most abundant and physiologically active form of vitamin E. Since the food content of tocopherols and tocotrienols is converted to α*-tocopherol equivalents*, the terms “vitamin E” and “α-tocopherol” are used interchangeably. The α-tocopherol, acting as a free radical scavenger preventing DNA oxidative damage, belongs to the antioxidant defense system. It notably protects polyunsaturated fatty acids (PUFAs) within plasma lipoproteins and membrane phospholipids, preserving thus the cellular membrane integrity (e.g., of erythrocytes, central and peripheral nerves). The α-Tocopherol has also been linked to cancer prevention through inhibiting cell proliferation and angiogenesis. The main dietary sources of α-tocopherol include vegetable oils and derivatives, some fatty fish, egg yolk, nuts, seeds, and whole grain cereals. The more abundant form in food are the α and γ-tocopherols (80).

### Metabolism of Vitamin E

As a lipid-soluble vitamin, upon intake, vitamin E follows intestinal absorption, hepatic and cellular uptake similar to those of lipids and other lipophilic components (81). About 75% of a usual α-tocopherol intake is absorbed by the human body and efficient absorption requires the presence of fat (80). Proteins involved in the vitamin E uptake (in the enterocyte) include the scavenger receptor class B member 1 (SCAR-BI), the Niemann–Pick disease type C1 protein (NPC1) and the cluster determinant 36 (CD36) molecule, also known as scavenger receptor class B member 3 (SRB3). All these also participate to the transmembrane transport of cholesterol and other lipophilic components (10, 22). Scavenger receptor class B member 1 (SCAR-B1) is a multi-ligand membrane receptor expressed in many cell types. It is not only involved in α-tocopherol uptake by enterocytes, but also in its transport from enterocytes to the blood, transfer of α-tocopherol from high-density lipoprotein (HDL) cholesterol to tissues and in the biliary excretion of α-tocopherol. The SCAR-B1 also acts as a plasma membrane receptor for HDL and mediates cholesterol transfer to and from HDL (11). The CD36 is a membrane glycoprotein involved in the uptake of fatty acids and in binding native and oxidized lipoproteins contributing thus directly or indirectly to the transport of vitamin E (22).

After a-tocopherol is absorbed in the intestine, it is integrated into chylomicrons and along the lymphatic system chylomicrons are secreted into circulation. Part of the a-tocopherol in chylomicrons is incorporated into tissues by lipoprotein lipase (LPL), while the remaining is transferred to the liver. In the liver, the α-tocopherol transfer protein (α-TTP) binds a-tocopherol with the highest affinity. The a-TTP is responsible for a-tocopherol incorporation into the preliminary very low density lipoproteins (VLDLs) which are secreted by the liver into the circulation and then distributed to body tissues. VLDLs are converted into intermediate-density lipoproteins (IDLs) and low-density lipoproteins (LDLs) by the action of LPL, and to HDLs (81). Plasma lipoproteins (VLDL, LDL, and HDL) are the major carriers of vitamin E. Thus, proteins involved in lipoprotein synthesis and metabolism play a crucial role in forming lipoproteins for vitamin E transport. In particular, apo-lipoproteins, LPL, hepatic lipase, phospholipid transfer protein (PLTP), cholesteryl ester transfer protein (CETP), and lecithin cholesterol acyltransferase are indirectly involved in vitamin E metabolism. Among the main apo-lipoproteins involved are apo A-IV and apo AV (10). Amounts of α-tocopherol not bound to α-TTP is catabolized in the liver by the hepatic enzyme cytochrome P (CYP)4F2 ω-hydroxylase. Both α-TTP and ω-hydroxylase have a significant contribution to α-tocopherol metabolism, especially to the liver balance (storage vs. catabolism) and to vitamin E systemic level (82).

### Biomarkers of Vitamin E Status

Fasting plasma or serum α-tocopherol concentration has been commonly used to assess vitamin E status. Since there is no precise cut-off value above which adequate status is characterized, plasma/serum α-tocopherol levels below 12 μmol/L may indicate deficiency. In α-tocopherol deficiency, oxidative stress can damage red blood cells (RBCs) (80).

a-Tocopherol concentrations within the range of 2.5–12 μmol/L have been described in primary or secondary deficiency (83, 84). Mutations in the a-TTP gene result in primary α-tocopherol deficiency and related neurological symptoms, including ataxia (85). Secondary a-tocopherol deficiency is present in individuals suffering from conditions including abetalipoproteinaemia, cholestatic liver diseases, severe malnutrition, or fat malabsorption (83, 86). Low a-tocopherol dietary intake has not been reported to cause deficiency with clinical manifestations in healthy individuals (80).

### Genetic Variations and Vitamin E Status

Vitamin E status is known to be affected by factors such as age, eating habits, oxidative stress (e.g., through smoking), absorption efficiency, and catabolism (10, 11). Genome-wide and candidate gene association studies have identified genetic variations that can influence vitamin E status, impairing its metabolism, absorption/uptake, transport and liver storage, or catabolism balance (11, 22). Borel and Desmarchelier (11) provide a comprehensive diagram illustrating the majority of genes encoding key players in the vitamin E status.

### Vitamin E Uptake Related Variants

There is strong evidence that SNPs in SCAR-B1 gene on chromosome 12q24.31 affect vitamin E metabolism and α-tocopherol levels (10, 11). Rs5888, which is also known as A350A, is a variant located on exon 8 of the SCAR-B1 gene including an exchange of the minor allele (C) for (T). TT carriers have the lowest plasma α-tocopherol levels as compared to CC or CT carriers and CT have shown the highest levels compared to the other two counterparts; these associations were significant (*p* < 0.05) in men after adjustment for cholesterol levels [mean a-tocopherol levels μmol/L (SD) per pair of alleles; TT: 23.47 (6.99) < CC: 26.14 (6.22) < CT: 28.07 (6.87)] (10). The Alpha-Tocopherol, Beta-Carotene Cancer Prevention (ATBC) Study was a randomized, double-blind, placebo-controlled intervention trial and evaluated cancer prevention after supplementation with either alpha-tocopherol, or beta carotene or both among male smokers (37). In a GWAS of serum α-tocopherol concentrations within the ATBC cohort, Major et al. (36) reported that the presence of the minor (A) variant allele of another SCAR-B1 SNP (rs11057830) was significantly associated with plasma α-tocopherol concentration (b = 0.04, *p* = 2.0 × 10^−8^) in male smokers independent of their age, BMI, cholesterol levels, and cancer status and with high response in serum concentrations to long-term a-tocopherol supplementation in the same population (b = 0.03, *p* = 2.9 x 10^−3^).

The CD36 SNPs might also influence plasma α-tocopherol concentrations. So far, two relevant SNPs (rs1761667 and rs1527479) have been identified and are found in high linkage disequilibrium. Individuals homozygous for the minor allele of rs1761667 (G) or rs1527479 (A) have lower plasma α-tocopherol concentrations than do carriers of the major allele (A of rs1761667, G of rs1527479) at about 22.9%, *p* = 0.046, and 23.7% *p* = 0.0061, respectively, especially among men after adjustment for age and BMI. The relationship between rs1527479 and a-tocopherol levels maintained its significance even after correction for multiple testing (*p* threshold < 0.0071). Furthermore, lower a-tocopherol levels were observed particularly among individuals carrying the A minor allele of rs1527479 with low triglyceride (*p* for trend = 0.013) or PUFA concentrations (*p* for trend = 0.005) than their corresponding GG individuals. The lower plasma a-tocopherol levels detected in individuals homozygous for the minor alleles may be related to a higher CD36 expression and a subsequent higher vitamin E and fatty acid transport. Dietary recommendations for higher vitamin E intakes among these individuals may be relevant to counteract the CD36 protein excess (35).

### Vitamin E Transport Related Variants

Apo-AV is a minor apo-lipoprotein almost exclusively expressed in liver that plays a significant role in the regulation of plasma triglycerides. The most studied SNP located on the Apo-AV promotor is rs662799 (1131T>C) in which the minor variant (C) has been associated with both higher plasma vitamin E and VLDL-TGs levels in diabetic patients. In particular, among TT carriers the mean (SD) vitamin E levels in blood were 40.32 mmol/L (10.47), whereas in TC carriers were 45.48 mmol/L (8.20) (*p* = 0.02). Moreover, plasma triglyceride (TG) concentration was 21% higher in carriers of the TC genotype (*p* = 0.04), because of higher TG in VLDL [mean TG (SD); 0.96 mmol/L (0.78) for TT carriers and 1.33 mmol/L (1.11) for TC carriers, *p* = 0.043] and in HDL [mean TG mmol/l (SD); 0.14 mmol/L (0.05) for TT carriers and 0.17 mmol/L (0.03) for TC carriers, *p* = 0.017) (87).

In a GWAS investigating the circulating α-tocopherol phenotype, Ferrucci et al. (25) reported that the rs12272004 SNP, close to the apo-AV gene, was associated with higher plasma α-tocopherol concentrations. In particular, the A allele of rs12272004 was associated with a 0.07 SD higher vitamin (95% CI = 0.05–0.10). When analysis was adjusted for TG levels, the strength of the association was reduced (b = 0.055, 95% CI = 0.020–0.091). Major et al. (36) identified a significant association between serum α-tocopherol and rs964184 on chromosome 11q23.3, within or near the gene cluster Apo-AI/CIII/AIV/AV. The rs964184 minor G variant allele was associated with increased a-tocopherol levels (b = 0.04, SE = 0.01, *p* = 2.7 x 10^−10^) after adjustment for age, BMI, non-HDL cholesterol concentrations, and cancer status (36) and with high serum response to long term vitamin E supplementation (b = 0.07, SE = 0.01, *p* = 2.6 x 10^−12^) (37).

Based on findings from a candidate gene association study, a variant of the apo A-IV was also found to be associated with vitamin E status. ApoA-IV is secreted in the intestine and is associated with chylomicrons. A polymorphism in the gene located on chromosome 11 result to an A to T substitution that changes the threonine residue at position 347 to serine ApoIVSer-347 (rs675). Women carrying the A allele (AT+AA) of rs675 were found to have significantly (*p* < 0.05) lower plasma concentrations of a-tocopherol than women homozygous for the T allele. [(mean a-tocopherol (SD) levels TT 34.12 μmol/L (8.68) > AA+AT 26.40 μmol/L (6.59)]. However, this association did not remain significant after adjustment for blood cholesterol levels (10). The same study confirmed the previously described association between plasma a-tocopherol and Apo-E variants. The human ApoE gene is polymorphic (derived from the combination of polymorphisms rs429358 and rs7412), which results in three major isoforms/alleles (ε2, ε3, and ε4), particularly the ApoE-ε2 (Cys112, Cys158), ApoE-ε3 (Cys112, Arg158), and ApoE-ε4 (Arg112, Arg158) alleles. Apo-lipoprotein E is a multifunctional protein involved in the catabolism of lipoprotein particles. In humans, the lowest plasma vitamin E concentrations were found in ApoE-ε2/ε2 genotype [mean a-tocopherol (SD); 18.68 μmol/L (8.45), *p* < 0.05], whereas the presence of the ε4 allele was associated with the highest vitamin E levels in plasma (ApoE-ε4/ε2 genotype) [mean (SD); 32.81 μmol/L (10.54) *p* < 0.05] (10).

Rare mutations in the a-TTP gene on chromosome 8q13 have been linked with severe vitamin E deficiency which cause an autosomal recessive neurologic disorder and ataxia in humans, also known as ataxia with isolated vitamin E deficiency (AVED) (85). Furthermore, the presence of the TT genotype of the rs6994076 TTPA polymorphism (980A/T) has been found to be associated with decreased protein activity and ~3% lower α-tocopherol levels (*p* = 0.03) compared to AA genotypes based on data collected among the control group of the ATBC trial (59). A lower (25%) response to vitamin E supplementation was observed in serum a-tocopherol concentration (*p* = 0.002, in multivariate model) in individuals (males) with the TT genotype compared to those homozygous for the major A allele, AA genotype (59).

As previously indicated, CYP4F2 encodes for cytochrome P450 4F2, which is involved in the vitamin E catabolism. The SNP rs2108622 on CYP4F2 gene results in a valine for methionine substitution. Subjects homozygous for minor T allele (TT genotype) were found to have reduced ω-hydroxylation activity and increased serum α-tocopherol (36). The TT genotype has also been significantly associated with increased serum response (b = 0.04, *p* = 2.2 x 10^−7^) to long-term (3 years) α-tocopherol supplementation, adjusted for age, BMI, non-HDL cholesterol concentrations, and cancer status (37).

## Vitamin C

Vitamin C or ascorbic acid is a water-soluble vitamin. Vitamin C acts as a free radical scavenger and operates as an enzyme cofactor for various biochemical reactions. Vitamin C is also involved in the biosynthesis of collagen, synthesis of carnitine and catecholamines and in the metabolism of cholesterol to bile acids. Vitamin C is naturally present mainly in fruit, vegetables, and potatoes. Exposition to oxygen or high temperatures affects its stability and results in vitamin C oxidation (88).

### Vitamin C Metabolism

Ascorbic acid (ascorbate) is the functional form of vitamin C. Two transporter proteins (SVCT1 and SVCT2) encoded by the sodium-dependent vitamin C transporter genes, SLC23A1 and SLC23A2, respectively, are responsible for vitamin's absorption by the gastrointestinal tract and reabsorption by renal system through active transport across membranes. Vitamin C concentrations and body homeostasis are regulated primarily by SVCT1 (89). The expression of SVCT1 in the kidney is important for the regulation of vitamin C status (41). The SVCT2 regulates vitamin C levels within specific metabolically active tissues (89). It promotes the accumulation of vitamin C into brain, eyes, and adrenals as it binds ascorbate with high-affinity (41). Dehydroascorbate, DHA, the oxidized form of ascorbate, is transferred into the cell by some glucose transporters, including GLUT1, GLUT3, and GLUT4. Within cells, DHA is recycled back to ascorbate, sustaining the intracellular ascorbate uptake (41). In plasma, the free anion of vitamin C is distributed to tissues. The body's absorption efficiency depends on the level of vitamin C intake. Vitamin C is excreted in urine, which is the main route of elimination, at an approximate proportion of 25% of the ingested amount for an intake of 100 mg/day (88).

### Biomarkers of Vitamin C Status

Ascorbate concentrations in plasma and leukocytes are considered suitable biomarkers of body stores and status, within the usual range of intakes and independently of recent vitamin C intake (88, 90).

A plasma ascorbic acid value of 50 μmol/L is indicative of an adequate status and values below 11 μmol/L indicate severe deficiency (biochemical and/or clinical symptoms including those related to connective tissue defects; scurvy). Plasma levels between 11 and 23 μmol/L (0.2–0.4 mg/100 mL) reflect marginal status, thus moderate risk of developing deficiency. The vitamin C physiological levels largely depend on the analytical methods used, limiting comparisons among laboratories. For instance, the interpretation of leukocyte vitamin C concentrations is complicated by the different concentrations of vitamin C in various leukocyte cell fractions—mononuclear contain up to 2- or 3-fold higher concentrations than polymorphonuclear cells (91).

### Genetic Variations and Vitamin C Status

Polymorphisms in the genes encoding sodium-dependent SVCTs of the SLC23 family are strongly associated with vitamin C status due to their roles in direct transport, absorption, and vitamin accumulation in tissues (41, 92). Genetic variants in protein-coding genes known to play a role in oxidative stress, including haptoglobin, glutathione-S-transferases, and manganese superoxide dismutase, may also influence vitamin C status, but they have not been a priori associated with ascorbic acid (41). A diagram outlining vitamin C metabolism and related genes is provided by Michels et al. (41).

In a cross-sectional study of 1.046 volunteers including Caucasian and East Asian populations, Cahill and El-Sohemy (15) reported that individuals may differ in their blood vitamin C levels, regardless of their dietary intake, due to genetic variation in SVCT1 (rs4257763). Overall, average serum vitamin C levels were lower in individuals with the GG genotype than the AA; with the GA genotype being intermediate (*p* = 0.002). In a sub-population analysis, differences remained but were significant only among Caucasian subjects (*p* = 0.02) and not among East Asians (*p* = 0.14).

Timpson et al. (53) performed a large-scale analysis (using a discovery cohort, the British Women's Heart and Health Study BWHHS and a series of follow-up cohorts and meta-analysis) to assess the relationship between variation at SLC23A1 (rs33972313) and circulating levels of L-ascorbic acid in over 15,000 Caucasian participants from five longitudinal studies. A pooled analysis of the relationship between rs33972313 (C/T) and vitamin C status across all (discovery and replication) studies showed that each additional minor allele (T) was associated with a reduction in plasma levels by 5.98 μmol/L (95%CI = −8.23, −3.73) per minor allele. The rs33972313 variant in SLC23A1 results in a valine to methionine substitution at position 264 of SVCT1 and in decreased transport activity. In the discovery cohort, two SNPs showed positive association with vitamin C status. For each additional minor allele of SNPs rs10063949 (T/C) and rs6596473 (G/C), there was an increase in circulating ascorbic acid levels [b = 1.91 μmol/L, 95% CI = 0.47–3.34 and b = 2.86 μmol/L, 95% CI = 1.39–4.33 per minor allele (C), respectively]. In the first stage replication study [the European Prospective Investigation of Cancer Norfolk Study (EPIC-Norfolk)], results were consistent with those found in the discovery cohort regarding the rs6596473 variant (mean difference in L-ascorbic acid = 1.01 μmol/L, 95% CI = 0.14–1.87, *p* = 0.02, per minor allele) but there was no association between rs10063949 and vitamin C status [mean difference in L-ascorbic acid = −0.05 μmol/L, 95% CI = (−0.90, 0.80), *p* = 0.9 per minor allele]. Among the polymorphisms examined, rs6596471 (A/G) in BWHHS showed a notable, albeit not significant, increase in plasma vitamin C levels [b = 0.95, 95% CI = (−0.63, 2.53) per minor G allele] (53).

In a case–control study, nested within the European Prospective Investigation into Cancer and Nutrition (EPIC) cohort of vitamin C transporter gene variants and gastric cancer risk (21), authors evaluated genetic variants of SVCT as predictors of vitamin C plasma concentrations in a subsample of participants. Authors applied a multiple linear regression model to assess associations between the SLC23A1 and SLC23A2 SNPs and the log-transformed plasma levels of vitamin C, adjusting for age, sex, country, smoking status, *H. pylori* infection, and season of blood collection. Two SNPs in SLC23A1 (rs33972313, rs11950646) and two SNPs in SLC23A2 (rs6053005, rs6133175) were statistically significantly associated with plasma vitamin C. In agreement with the findings of Timpson et al. (53), being a heterozygote (GA) in the rs33972313 SNP in this study was associated with lower plasma vitamin C levels, (GA vs. GG or AA in a codominant model, b = −0.28, 95% CI = −0.54, −0.016). Variant rs11950646 was also associated with lower plasma vitamin C, in a dominant model [GG or AG vs. AA, b = −0.14, 95% CI = (−0.26, −0.011)] and per G allele in an allelic or log-additive model [b = −0.11, 95% CI = (−0.20, −0.017)]. In the case of the SLC23A2 gene, higher plasma vitamin C concentrations were associated with rs6053005 TT homozygotes b = (0.21, 95% CI = 0.058–0.37) and rs6133175 GG homozygotes (b = 0.22, 95% CI = 0.029–0.40) in a recessive model (21).

Another SNP in SLC23A2 gene (rs1279683, A>G) was found to be significantly associated with plasma concentrations of vitamin C. In a case-control study including patients of primary open-angle glaucoma (POAG), homozygous subjects for the G allele (GG) had significantly lower plasma vitamin C levels than the other genotypes (AA + AG) (9.0 ± 1.4 vs. 10.9 ± 1.6 μg/mL, *p* < 0.001 in cases and 10.9 ± 1.6 vs. 12.1 ± 1.8 μg/mL, *p* < 0.001 in controls) even after multi-variate adjustment for sex, age, BMI, smoking and alcohol, and after applying Bonferroni corrections for multiple comparisons (61). Unlike SLC23A1, genetic variation in SLC23A2 has little influence on vitamin C homeostasis and status, but controls the accumulation in tissues. Furthermore, studies are also lacking since most of the SNPs studied to date are located in intronic or untranslated regions and do not directly affect the SVCT2 protein coding (41).

Haptoglobin is a hemoglobin-binding protein, participating in iron metabolism and encoded by a polymorphic gene (haptoglobin gene, Hp) located on chromosome 16. In humans, Hp is characterized by a polymorphism with two alleles (Hp1 and Hp2) forming three main phenotypes: homozygous for the Hp1 allele (Hp 1-1 phenotype), heterozygous (Hp 2-1) and homozygous for the Hp2 allele (Hp2-2). Unlike Hp1, the hemoglobin protein derived from Hp2 binds insufficiently to hemoglobin and individuals expressing the Hp2-2 phenotype (Hp2-2 homozygotes) produce a less active protein and have increased circulating levels of iron compared to Hp1-1 (41). As a result, Hp 2-2 individuals are more susceptible to vitamin C deficiency since their plasma ascorbate is less stable (93). Na et al. (43) investigated the relation between haptoglobin genetic variants, vitamin C, and iron status in 110 Chinese subjects and reported that serum vitamin C was lower in Hp2-2 healthy Chinese male participants compared to both Hp2-1 and Hp1-1 individuals (*p* = 0.028). In the same study, vitamin C was also affected by iron status (ferritin levels) (43). It is however uncertain whether variations in vitamin C status related to haptoglobin polymorphism associate with iron dysregulation (affected by low dietary iron intake or anemia) especially among Hp2-2 individuals (41).

According to Cahill and Sohemy (16) individuals homozygous for the Hp2 allele had lower vitamin C concentrations compared to those with the Hp1 allele when their dietary vitamin C intake was low. In a cross-sectional examination of free-living adults enrolled in the Toronto Nutrigenomics and Health Study, individuals with the Hp2–2 genotype who did not meet the US Recommended Dietary Allowance for vitamin C had a significantly increased risk of ascorbic acid deficiency <11 μmol/L (adjusted OR = 4.77, 95% CI = 2.36–9.65) compared to those who did. Conversely, the risk of deficiency for carriers of the Hp1 allele (Hp2-1 + Hp1-1 phenotype) was lower and not significant (OR = 1.69, 95% CI = 0.80–3.63) after adjusting for BMI, sex, energy intake, use of oral contraceptives, C-reactive protein levels, plasma α-tocopherol, ethnicity, and season (16). Based on this evidence, Schwartz (46) concluded that the required daily vitamin C intake in Hp 2-2 individuals should be higher than other Hp phenotypes.

The glutathione S-transferases (GSTs) are partof a large family of enzymes involved in the detoxification of detrimental compounds. Genetic variations in GST may affect vitamin C status through their impact on reactive oxygen species and glutathione status. Deletion polymorphisms in the GSTM1 and GSTT1 genes, found on chromosome 1 and chromosome 22, respectively, are generally present in Caucasian populations and result in absence of enzyme function (41) Subjects with the GSTM1-null genotype appeared to have increased vitamin C concentrations compared with carriers of the functional gene variant (8). Horska et al. (30) reported lower plasma vitamin C levels in subjects with deletion of GSTM1 compared with subjects carrying the functional gene variant (*p* = 0.042). In the cross sectional evaluation of participants in the Toronto Nutrigenomics and Health Study, however no differences in vitamin C status were observed (14). GSTM1 genotype and vitamin C status may be characterized by a more complex relation, taking into account dietary vitamin C intake and other environmental exposures such as smoking (41).

Dietary intake, GST genetic variants and serum ascorbic acid concentrations were determined for about 900 non-smoking men and women. When compared to individuals who met the Recommended Dietary Allowance for vitamin C, individuals who did not adhere to vitamin C recommendations and possessed the GST null genotypes had an increased risk of vitamin C deficiency. The odds ratio (95% CI) for deficiency among individuals with the GSTM1 null (0/0) genotypes and functional GSTM1 (1/1+1/0) not meeting the RDA values for vitamin C were 4.03 (2.01, 8.09) and 2.29 (0.96, 5.45), respectively as compared to those with intakes in accordance to recommended levels. For GSTT1 variant, the odds ratio (95% CI) for deficiency were 2.17 (1.10, 4.28) for functional and 12.28 (4.26, 33.42) for null genotypes who did not comply with recommendations as compared to those who did (14). Individuals with GSTM1/GSTT1 deletion (null genotypes) and low intake of vitamin C from their diet had lower plasma vitamin C concentrations than those with functional enzymes at the same level of intake. This may suggest that these enzymes protect against deficiency when the vitamin C intake is insufficient (14, 30). Cahill et al. (14) also reported that subjects with the GSTT1-null genotype had decreased serum ascorbate. However, these findings were not confirmed by Block et al. (8), in which no statistically significant association was observed (*p* > 0.05). A loss of GSTT1 function appears to affect cellular ascorbate levels but not plasma concentrations compared to other GSTs (41). In general, associations between vitamin C and glutathione S-transferases are complex and not well-understood yet.

The superoxide dismutase (SOD) is considered a significant antioxidant enzyme in the body and exists in three SOD isoforms including the mitochondrial SOD manganese dependent (MnSOD), encoded by the gene SOD2 located on chromosome 6q25, in humans. A common SOD2 polymorphism, the Ala16Val (rs4880) results in a mutation at codon 16 and an alanine-to-valine substitution (GCT->GTT). The Val variant may be present at a lower concentration in the mitochondria and individuals homozygous (Val/Val) may have decreased resistance to oxidative stress (41). Duarte et al. (20) have studied the association of MnSOD variants with plasma vitamin C levels. Healthy homozygous for the valine variant individuals have higher levels of serum vitamin C (*p* < 0.05). In contrast, subjects with hypercholesterolemia and at least one copy of the val variant had lower serum vitamin C concentrations compared to subjects homozygous for the alanine variant, indicating an increase in the oxidative stress in subjects with the VV genotype for MnSOD and hypercholesterolemia (20).

## Folate and Vitamin B12

From the group of B vitamins, folate (vitamin B9) and cobalamin (vitamin B12) have been primarily studied in relation to genetic variants affecting their body status. Therefore, this section focuses on the presentation of evidence related to these two B-vitamins. Folate is used to collectively describe a family of water-soluble compounds which are included in the B-complex vitamins. They are essential co-factors in one-carbon metabolism pathway (94), which involves three major interrelated metabolic cycles in the cells' cytosol and is essential in multiple biochemical processes, including: (a) amino acid metabolism and homeostasis, (b) *de novo* nucleotide synthesis (purines and thymidine; precursors for DNA and RNA), and (c) the process of methylation. Furthermore, the folate pathway is closely related to homocysteine metabolism (95, 96). Vitamins B12 and B6 (pyridoxine) are also important enzyme cofactors or substrates in one carbon metabolism (94).

Folate is present in high amounts in dark green leafy vegetables, legumes, orange and grapefruit (juice), and nuts (peanuts, almonds). Although folate in meat is generally found in low amounts, liver and kidney are particularly rich sources. Dairy products, fish, eggs, and potatoes are also sources of folate intake (96). Principal sources of cobalamin include products of animal origin (meat, fish, dairies, eggs, and liver) and it can also be added to foods and food supplements (97).

### Metabolism and Functions of Folate and Vitamin B12

**Dietary folate** mainly exists as polyglutamates, which are hydrolyzed to monoglutamates by folyl poly-γ-glutamate carboxypeptidase (FGCP) found in the intestinal mucosa. In the intestinal cells, folate is usually reduced and methylated to be absorbed in the small intestine by the high-affinity proton-coupled folate transporter (PCFT1). In the circulation, the prominent form of folate is 5-Methyl-tetrahydrofolate (5-methyl-THF) monoglutamate. About 50% of folate is bound to albumin, in plasma about one-third is in a free form and a small proportion is bound to the plasma folate receptor. Folate molecules enter the cell through folate receptors (FRs) via endocytosis, with FR-α having a high affinity for the monoglutamate 5-methylTHF (42).

The 5,10-methylenetetrahydrofolate (5,10 methyl-THF), one of the one-carbon substituted forms of tetrahydrofolate present in the cell, plays a central role in the folate and methionine cycles, as it can be used for thymidylate synthesis, or converted to 5-methyl-THF in the methionine synthesis cycle, or oxidized to 10-formyl-tetrahydrofolate for purine synthesis. The reduction of 5,10 methyl-THF to 5-methyl-THF is achieved by the methylenetetrahydrofolate reductase (MTHFR), which is a riboflavin-dependent enzyme. In the methionine cycle, 5-methyl-THF is used by methionine synthase (MTR) for the vitamin B12-dependent conversion of homocysteine to methionine and the formation of tetrahydrofolate (THF). Methionine synthase reductase encoded by MTRR gene is required for the reactivation and proper function of MTR (47).

**Vitamin B12** in food is bound to proteins and is released in the stomach under the influence of hydrochloric acid and pepsin. The thus released vitamin initially binds to dietary proteins, including haptocorrin (transcobalamin I, TC-I). Cobalamin is released from its complex with TC-I by pancreatic enzymes in the duodenum and then free vitamin binds to gastric intrinsic factor (IF), a glycoprotein produced in the stomach. After IF-cobalamin complex is absorbed through receptor-mediated endocytosis in the lower part of small intestine, cobalamin is released into the blood stream primarily in the form of methyl-cobalamin and the IF is degraded in lysosomes. A protein called transcobalamin II (TC-II) combines with cobalamin to form holotranscobalamin-holoTC (metabolic active cobalamin) and the complex is transferred to the cells after binding to the transcobalamin receptor TC-R. The vitamin's dietary source, its ability to be released from food and bind appropriately to IF determine cobalamin's absorption. The main fraction of plasma cobalamin (70–90%) binds to haptocorrin, while holoTC accounts for 10–30% of total plasma levels (47, 97).

Vitamin B12 is involved in the cytosolic transmethylation of homocysteine to methionine by the enzyme methionine synthase. Cobalamin is also required as coenzyme to form succinyl coenzyme A (CoA) from methylmalonyl CoA in propionate metabolism by methylmalonylCoA mutase in mitochondria (97).

### Biomarkers of Status

A sensitive and short-term indicator of folate dietary intake is serum or plasma folate concentration (98), while red blood cell (RBC) folate concentration is a marker of long-term intake responding gently to changes—and the most reliable biomarker of status, as it indicates tissue folate stores (96). The assessment of folate status should include either multiple measurements of serum/plasma folate over several weeks or a single measurement combined with other biomarkers of status (RBC folate). This combination is recommended by a World Health Organization (WHO) Technical Consultation on folate and cobalamin deficiencies, at the population level (96, 99).

**Folate** adequacy is described by serum/plasma and red blood cell folate concentrations of higher than 10 nmol/L (4.4 ng/mL) and 340 nmol/L (150 ng/mL), respectively. Serum folate and RBC concentrations of less than 6.8 and 317 nmol/L, respectively, are indicative of folate deficiency. Folate deficiency adversely affects DNA replication and synthesis and thus cell division. As a result, bone marrow may produce uncommonly large cells with abnormal maturation of nuclei and development of megaloblastic anemia (96). Furthermore, folate deficiency impairs the methionine cycle functions, resulting in elevated plasma homocysteine and insufficient production of the S-adenosylmethionine (SAM) which is extensively used in methylation reactions (100). The association of folate status during pre-conceptional period and appearance of developmental anomalies, including neural tube defects (NTD), has long been established (101).

Serum **cobalamin** is the most widely used biomarker of vitamin B12 status, reflecting both the metabolically active cobalamin bound to TCII and the inert fraction bound to haptocorrin. However, since its concentration associates weakly with the biomarkers of cobalamin function, serum holoTC is the most specific biomarker for assessment of adequate vitamin B12 status. Other biomarkers studied (e.g., methylmalonic acid and homocysteine) are elevated in cobalamin deficiency but are influenced by numerous dietary and lifestyle factors or conditions (in poor renal function, for instance) (99, 102). Thus, their interpretation requires a more complete assessment of the body nutritional status (99).

In clinical settings, serum total **vitamin B12** is used to assess adequacy, with serum total B12 concentrations below 148 pmol/L indicating deficiency. Notwithstanding the limitations indicated in the previous section, the concentrations of homocysteine and methylmalonic acid in blood have also been used to assess vitamin B12 status, with homocysteine levels above 15 μmol/L and methylmalonic acid above 750 nmol/L frequently used in adults to diagnose cobalamin deficiency. Clinical cobalamin deficiency is most frequently associated with megaloblastic anemia, as well as with neurological symptoms (97).

### Genetic Variations and Folate Status

Several polymorphisms in genes encoding enzymes and transport proteins of folate metabolism are reported to affect folate status, homocysteine levels, and health outcomes. Folate-mediated one-carbon metabolism and related genes have been illustrated (101). To date, most studies have reported that the MTHFR C677T (rs1801133) SNP is related to both biomarkers of status and disease risk (54, 103–105). Together with rs1801131, they are the most well-known genetic factors influencing folate status that have been studied in great detail in terms of molecular mechanism and impact on disease risk (29, 42). However, the 1298A-C MTHFR variant (rs1801131), which is in strong linkage disequilibrium with the 677C-T variant, has no effect on biomarkers of folate status (42, 48). The distribution of the polymorphism shows a substantial variation worldwide and across ethnic groups. The frequency of the MTHFRrs1801133 TT SNP is described to be high in Europeans, Asians, Central, and South Americans (10–32%) and low in several African populations (0–3%) (106). Binia et al. (107) reported that the TT genotype presents at about 25 and 57% in Mexican Mestizo and American-Indian populations, respectively.

The common rs1801133 variant is a C to T transition at position 677, resulting in substitution of alanine with valine. MTHFR is a flavoprotein incorporating loosely bound flavin adenine dinucleotide (FAD). This substitution results in weaker binding affinity for the riboflavin (vitamin B2) cofactor and increased loss of the FAD cofactor, creating a mildly dysfunctional thermolabile protein (108). Homozygosity for the T allele (TT genotype) is associated with reduced enzyme activity (109), lower serum and RBC folate and higher plasma homocysteine levels compared with the 677CC (31, 110, 111). The well-documented association between MTHFR rs1801133 and folate status has also been evaluated in more recent studies (13, 18, 48).

In their meta-analysis of the association between the MTHFR rs1801133 polymorphism and blood folate concentrations (plasma/serum, RBC), Tsang et al. (54) reported a steady difference in serum/plasma and RBC folate concentrations across MTHFR rs1801133 genotypes showing a distinct pattern of CC > CT > TT. The percentage difference was highest for the CC vs. the TT genotype [S/P = 13%, 95% credible interval (CrI) derived from Bayesian statistics = 7–18%] [RBC = 16%; 95% CrI = 12–20%] followed by CC vs. CT (S/P = 7%; 95% CrI = 1–12%] [RBC = 8%; 95% CrI = 4–12%) and CT vs. TT (S/P = 6%; 95% CrI = 1–11%] [RBC = 9%; 95% CrI = 5–13%]. The inheritance of one recessive allele (i.e., the CT genotype) is related to concentrations intermediate to the CC and TT genotypes, as described by the additive model (54).

Hyperhomocysteinemia is known to be most pronounced if the TT genotype occurs in combination with low nutritional status of either folate (110) or riboflavin (31, 39). Several studies have reported that individuals with the homozygous TT genotype exhibit decreased response of folate biomarkers to folate intervention compared to those with the homozygous CC genotype, suggesting a higher requirement for folate (29). The EFSA NDA Panel has taken this polymorphism into account in setting the dietary reference values for folate, applying a coefficient of variation of 15% to address additional variability (96). Moreover, genetically at-risk adults (TT homozygotes) also have higher riboflavin requirements in order to maintain adequate enzyme function (39, 40). Among TT genotypes, riboflavin supplementation results in decreased homocysteine concentrations by 22% overall (*p* = 0.03) and 40% among individuals with lower riboflavin status at baseline (*p* = 0.010) (40).

### Genetic Variations and Vitamin B12 Status

In a recent publication, Surendran et al. (51) reviewed candidate gene studies and GWAS published until May 2017 and conducted primarily among Caucasian populations, in order to identify associations between SNPs in genes related to vitamin B12 pathway and their impact on the circulating cobalamin levels. Authors present a comprehensive description together with schematic presentations of SNPs in genes related to co-factors, regulators of the vitamin transport, membrane cobalamin transporters or factors involved in enzymatic reactions of the one-carbon cycle (e.g., MTHFR and MTRR).

In their review, Surendran et al. (51) present, among others, the fucosyltranferase 2 gene. The FUT2 or secretor (Se) gene is located on chromosome 19 coding the a(1,2) fucosyltransferase enzyme that is involved in the production of H antigens (common precursors for the blood group A and B antigens expressed on secretory glands and digestive mucosal surfaces) and may also influence B12 absorption at the gastric level. In a meta-analysis of studies among US Caucasian populations Hazra et al. (27) concluded that the SNP rs601338, also known as 428 G/A non-secretor variant, was significantly associated with plasma vitamin B12 concentrations. In particular, the A allele was positively associated with circulating vitamin B12 levels (β = 0.06 pg/mL, SE = 0.01). There is a heterogeneity in associations between FUT2 polymorphism and cobalamin concentrations, as the distribution of the minor allele of rs601338 considerably varies among ethnicities. Hazra et al. (27) reported that the rs492602 SNP is in complete linkage disequilibrium with rs601338. The G allele of the SNP rs492602 variant was associated with lower vitamin B12 levels (β = 0.06 pg/mL, SE = 0.01) Interestingly, in another study among 3114 Canadian adults, the G allele of the same SNP was found to be associated with a lower risk (OR = 0.60, 95% CI = 0.54–0.70) of vitamin B12 deficiency (<148 pmol/L), compared to A allele (62). The most commonly studied FUT2 variant however is the rs602662 SNP, which is also reported to be in linkage disequilibrium with the rs601338 SNP. Carriers of the A allele in the rs602662 variant are at lower risk (OR = 0.61, 95% CI = 0.47–0.80) of vitamin B12 deficiency (<148 pmol/L), compared to those carrying the G allele (62). Genetic variations in the FUT2 gene have also been implicated in alterations of the composition of the gut microbiome and individuals with the non-functional FUT2 phenotype (non-secretors) are susceptible to Helicobacter pylori infection and subsequent vitamin B12 malabsorption (51).

Another variant described in the Surendran et al. (51) review is the transcobalamin 2 (TCN2) gene, located on chromosome 22, which encodes transcobalamin II (vitamin B12 binding protein). The most commonly reported association between TCN2 polymorphism and vitamin B12 levels in Caucasians is the SNP rs1801198, characterized by C to G substitution at nucleotide 776 (TCN2 776C>G) and an exchange of proline to arginine at codon 259. In a candidate gene association study among 613 Irish men (50), individuals with the TCN2 776CC genotype were associated with lower serum vitamin B12 levels compared to those with 776 CG (adjusted *P* = 0.03) and 776GG genotypes (adjusted *P* = 0.045). In contrast, in a cross-sectional study of 122 individuals from Portugal, holotranscobalamin (Holo-TC) levels were significantly associated with the SNP rs1801198; carriers of the G allele had lower Holo-TC concentrations than C carriers (*P* < 0.05) (17), a finding which has also been reported in an earlier cross-sectional study (57).

Cubulin (CUBN), CD320 and methionine synthase reductase (MTRR) genes are also vitamin B12 related genes described in the Surendran et al. (51) review. CUBN is known as the intrinsic factor-cobalamin (IF-B12) receptor located on chromosome 10 and variants within this gene have been associated with B12 status, but results are often conflicting. Hazra et al. (27) reported that the A allele of the rs1801222 (Ser253Phe) variant was associated with lower cobalamin status (β = −0.05 pg/mL, SE = 0.01) in 4763 US individuals, while subjects homozygous for the rs1801222 G allele had higher vitamin B12 concentrations. In contrast, Zinck et al. (62) described that the G allele of the rs1801222 was associated with an increased risk of cobalamin deficiency (OR = 1.61, 95% CI = 1.24–2.09).

The CD320 or “CD320 molecule” gene encodes the transcobalamin receptor (TCblR) and is located on chromosome 19. The most commonly studied variant is the rs2336573 variant characterized by a glycine to arginine change, at codon position 220. Zinck et al. (62) reported that the C allele of this variant was associated with a lower risk (OR = 0.62, 95% CI = 0.45–0.86) of inadequate vitamin B12 levels (<220 pmol/L). An earlier study among Icelandic and Danish individuals (*n* = 45,571 adults), however, reported that the “T” allele was associated with increased vitamin B12 concentrations (effect β = 0.22–0.32 pmol/L; *P* = 8.4 × 10^−59^) (23).

Genetic variants in methionine synthase reductase (MTRR) gene, located on chromosome 5, have also been associated with vitamin B12 levels in healthy individuals. Among them, SNP MTRR rs162036 (Lys350Arg) was found to be associated with vitamin B12 levels in a study of 2424 twins of North European descent (Caucasians) (*p* = 0.04) (4).While the majority of candidate gene association studies did not provide evidence (*P* > 0.05) for an association between the MTHFR gene polymorphisms (rs1801131 and rs1801133) and cobalamin concentrations, Thuesen et al. reported that the TT genotype of the rs1801133 variant was associated with a higher risk of vitamin B12 concentrations below 148 pmol/L compared with the CC genotype (OR = 1.78, 95% CI = 1.25–2.54) in a study among Danish participants (52).

## Discussion

We have undertaken a comprehensive narrative review of the literature available until March 2020 in order to identify genetic variants extensively studied that could impair the body's vitamin status. The fat-soluble vitamins D and E and the water-soluble vitamins C, B9 (folate), and B12 (cobalamin) prevail in the corresponding literature and have therefore been presented in this review. Based on the outcomes of candidate gene studies, GWAS and the meta-analysis thereof, several SNPs have been identified to impair enzymes, carrier proteins, cell membrane channels and similar routes and substances that can subsequently affect the absorption, release in the blood stream and cell uptake of the vitamins under study. Therefore, through possible impacts on the vitamins' physiology, metabolism, and functionality, several of these genetic variants could determine the individual's vitamin status following an intake through the diet. Evidence is however not always consistent and we opted for the presentation of both converging and diverging results which could be attributed to either the heterogeneity among the populations studied, lack of comparability between methods and different biomarkers of vitamin status or a combination of all.

Furthermore, for the vast majority of these polymorphisms, prevalence rates in the general population are not known and cannot be used for setting advice on dietary requirements at the population level or design public health actions. An exception, however, holds for the European dietary reference values for folate in which the MTHFR genotype (rs1801133) has been taken into consideration. In particular, after setting the Average Requirement for folate, the EFSA Panel of Nutrition, Novel Foods and Food Allergens (96) considered evidence on the prevalence of the various genotypes in the European population and on the higher folate requirements among individuals with the MTHFR 677TT, compared to those with the MTHFR 677CC genotype, and selected a larger coefficient of variation to be applied in setting the Population Reference Intake.

Next to their impact on vitamin status, several of the polymorphisms presented have also been associated with disease risk. Variants in the vitamin D-related VDR gene have commonly been associated with the risk of a range of skeletal and non-skeletal health outcomes, including infections from the respiratory syncytial virus, the hepatitis B virus; tuberculosis; cancer, and autoimmune diseases such as systematic lupus erythematosus and rheumatoid arthritis (32, 112). Polymorphisms in the SLC23A1 and SLC23A2 genes as well as in other genes encoding the vitamin C transporter have also been associated with the risk of chronic diseases, including cancer, inflammatory bowel disease, preterm delivery, coronary heart disease, and glaucoma (113).

Several studies indicated gene-diet interactions between folate intake, MTHFR C677T genotype and risk of breast, colorectal, and lung cancer (114). With respect to colorectal cancer, meta-analyses of case-control studies provided evidence that the 677TT genotype is associated with a lower risk of colorectal cancer when compared to the CC genotype (105, 115). In another meta-analysis of case-control studies including CHD patients, individuals with the 677TT genotype had a significantly higher risk of CHD (OR = 1.16, 95% CI = 1.05–1.28) compared to the CC genotype, particularly when combined with low folate status (OR = 1.44, 95% CI = 1.12–1.83) (116). Cronin et al. (104) reported a dose-response association between the MTHFR 677T allele and the risk of ischemic stroke (T allele pooled OR = 1.17, 95% CI = 1.09–1.26 and TT genotype pooled OR = 1.37, 95% CI = 1.15–1.64, respectively). Homozygosity for the MTHFR C677T polymorphism (TT genotype) has also been associated with an increased risk for NTD-affected pregnancies (103), which provides additional support to the well-reported link between folate status and NTD risk. The presentation of such gene-disease associations were out of the scope of the present manuscript.

Variants related to vitamin E transport have also been associated with disease risk. Apo-AV is a minor apo-lipoprotein almost exclusively expressed in liver that plays a significant role in the regulation of plasma triglycerides. The most studied SNP in Apo-AV is rs662799 where the minor variant (C) has been associated with both higher plasma vitamin E and VLDL-TGs levels in diabetic patients. Guardiola and Ribalta (117) reported that in patients with metabolic syndrome or type 2 diabetes, apo-AV variants increase TG levels and minor allele carriers present an altered lipoprotein profile including large VLDL and small LDL and HDL particles that characterize atherogenic dyslipidemia.

Some considerations are crucial when reporting and interpreting genetic studies in relation to vitamin status. Confounding generated through the use of less appropriate comparators can lead researchers to spurious findings. Ethnicity and race are for instance important characteristics that can confound the associations observed (118). In our review, we generally selected to present studies that controlled for self-reported ethnicity. Misclassification due to either the biomarker of vitamin status used or the analytic validity of genetic markers is an additional issue to consider. As regards the vitamin-related biomarker, we have solely relied on studies presenting associations between genetic variants and conventional biomarkers of vitamin status that have been widely used by national and international scientific bodies (64, 68, 80, 88, 90, 96, 97). With respect to the validity of the genetic markers used in the studies we report in this review, unless errors are systematic, any misclassification would have probably attenuated the association between a dichotomous biomarker and the risk of low vitamin status, but it would probably not generate an association when in reality this does not exist. Studies of genetic epidemiology making use of numerous gene variants suffer from an increased possibility for Type I and Type II errors, particularly when authors overemphasize on statistical testing. In our presentation of results, we selectively described the outcome of methods that addressed the problem of false positive associations resulting from multiple comparisons (e.g., *p*-values after the application of Bonferroni correction or Bayesian methods, when available). Next to statistical significance, biological relevance needs to be also taken into account in combination or as a separate criterion to establish causality.

The strengths of our narrative review lie in the provision of a collective description of converging and diverging evidence regarding genetic variants that influence vitamin status in response to dietary intake. Furthermore, our review summarizes studies among individuals from the general population, free of prevalent diseases that could affect their nutritional status addressing thus issues related to the public health. Our review is limited by the fact it focuses on vitamin deficiencies. However, it should be pointed out that the excess of water-soluble vitamins is physiologically controlled through homeostatic mechanisms and health professionals are primarily concerned with the deficiency rather than the excess of lipo-soluble vitamins (vitamins D and E, in our case).

The ongoing miRNA research is providing additional evidence on gene expression, body function and many other biological processes. miRNAs have been reported to target vitamin-related genes and human circulating vitamin levels (119). Nevertheless, the currently published research primarily explores such associations among patients of degenerative diseases/conditions. The demonstration of possible interactions among free-living, generally healthy individuals is yet to be accumulated and will substantially enhance our knowledge on disease progression.

In conclusion, our review summarizes current findings on how genetic variants could shape inter-individual differences in response to dietary intakes. Capturing these differences in intakes and mitigate sources of bias that could confound any association observed has traditionally been the goal of objective dietary assessment studies. The consideration of genetic variants has its own share in improving our understanding of the diet-disease inter-relationship. The individual genetic profile could add an extra layer of personalization in nutritional evaluation and offer a more comprehensive dietary assessment. Next to their association with vitamin status, genetic variants have also been implicated in underlying needs for differential vitamin intake to prevent chronic, diet-related diseases. The early knowledge of those needs could drive personalized nutritional advice favoring prevention. Well-designed studies addressing biological relevance next to statistical significance are however needed if the identification of genetic variants it to be included in future dietary assessments.

## Author Contributions

ANi performed the literature review, in collaboration with VK. ANi drafted the paper. All authors contributed in the development of the submitted manuscript. ANa has the primary responsibility for the manuscript's final content.

## Conflict of Interest

VK is the founder of MEDOLIALI SL, a nutrigenetic consulting and supporting SME. The remaining authors declare that the research was conducted in the absence of any commercial or financial relationships that could be construed as a potential conflict of interest.
